# Transfer of training through institutional partnership: outcomes from a faculty development program in China

**DOI:** 10.1186/s12909-026-09726-2

**Published:** 2026-07-09

**Authors:** Jonathan Lio, Helen Barrett Fromme, Yue Li, Hongmei Dong, Zhanghong Lu, Jingyi Fan, Yanqing Ye, Ivy Jiang, Renslow Sherer

**Affiliations:** 1 Department of Medicine, University of Chicago Pritzker School of Medicine, Chicago, USA; 2 Department of Pediatrics, University of Chicago Pritzker School of Medicine, Chicago, USA; 3 Department of Gatroenterology, Peking Union Medical College Hospital, Chinese Academy of Medical Sciences & Peking Union Medical College, Beijing, China; 4 Teaching Office, Renmin Hospital of Wuhan University, Wuhan, China; 5 Department of Pediatrics, Zhongnan Hospital of Wuhan University, Wuhan, China; 6 Department of Medicine, Zhongnan Hospital of Wuhan University, Wuhan, China

**Keywords:** Faculty development, Program evaluation, Transfer of training, Institutional partnership

## Abstract

**Background:**

International faculty development programs aim to strengthen teaching capacity, yet their impact depends on whether participants can apply new skills in their own institutions. In China, few studies have examined outcomes from multi-institutional programs and the process by which institutional partnerships support application of learning. This study evaluates the International Medical Educators Program (IMEP), a longitudinal faculty development program in China focused on curriculum development, teaching skills, and educational leadership, and examines its medium- to long-term outcomes.

**Methods:**

A cross-sectional survey was administered in 2025 to all IMEP participants from 2019–2024 (*n* = 86), representing 1–5 years post-training. The survey assessed Kirkpatrick level 3 and 4 outcomes, including project implementation, dissemination, educational impact, and professional advancement. Outcomes were compared across institutional groups using chi-square tests and multivariable logistic regression.

**Results:**

Sixty alumni completed the survey (69.8%). Overall, 60% implemented their original project, 63.3% initiated new teaching projects, 75% shared IMEP learning with colleagues, and 23.3% presented regionally or nationally. Level 4 outcomes included: 50% received a teaching award, 25% achieved promotion based on educational work, 26.7% assumed new medical education leadership roles, 50% led curricular reform, and 48.3% published educational work. After adjusting for faculty seniority, only promotion remained significantly associated with institutional affiliation (aOR 4.88, 95% CI 1.06–22.51).

**Conclusions:**

IMEP demonstrates that longitudinal, partnership-based faculty development can be associated with sustained outcomes across diverse institutional contexts in China. Site visits, ongoing mentorship, and continued engagement through institutional partnerships may help explain sustained application of learning beyond the training period. Despite institutional differences, faculty from all hospitals achieved substantial impact, highlighting the value of inclusive approaches to building capacity in medical education.

## Background

Faculty development is critical to improving the quality of medical education and healthcare worldwide in order to maintain currency, consistency, and innovation among faculty [5, 11]. Longitudinal programs that incorporate mentoring, experiential learning, and project work can produce sustained behavioral and organizational outcomes, including new educational initiatives, increased academic productivity, and new leadership positions [17].

Notable national and international training programs have illustrated the potential of this approach. The Josiah Macy Jr. Foundation’s Faculty Scholars Program has demonstrated the value of protected time, mentoring, and strong communities of practice [8], while the FAIMER Institute has shown that longitudinal, project-based programs can produce measurable impact at multiple levels [20]. However, most published evidence on longitudinal faculty development programs comes from North American and Western European settings, limiting generalizability to rapidly evolving contexts such as China. The limited evidence from Asia [9] and small number of studies from China [3, 21] highlight an important gap.

China presents a particularly important setting in which to examine faculty development. Over the past decade, major national reforms have transformed residency training and sought to improve teaching quality [12]. Despite these reforms, however, many institutions continue to report limited faculty preparation in educational theory and teaching practice. Addressing these needs requires not only technical training but international partnerships that help faculty adapt educational practices to their local institutional contexts.

The longstanding collaborations between the University of Chicago and its partner hospitals in Wuhan and Beijing exemplify such partnerships. These collaborations have included curriculum reform, faculty development initiatives, and academic exchange, demonstrating the feasibility of long-term educational engagement across institutions [15]. Building on this foundation, the International Medical Educators Program (IMEP) was launched in 2019 as a faculty development program organized by the University of Chicago with Wuhan University-affiliated hospitals and Peking Union Medical College Hospital (PUMCH).

IMEP’s design is informed by transfer-of-training theory. Transfer of training refers to the extent to which learning from a program is applied in the workplace, a central outcome for faculty development initiatives. Baldwin and Ford’s seminal model describes three factors influencing transfer: trainee characteristics, training design, and work environment [2]. While most faculty development programs focus on the first two factors, organizational factors are harder to address, particularly when participants return to their institutions outside the program’s direct influence [4]. IMEP’s design and partnership model were intended to engage the work environment through supervisor involvement, institutional accountability, and post-training support for project implementation.

Despite increasing attention to faculty development in China, few published studies examine outcomes of longitudinal programs in China and the manner in which contextual factors such as institutional setting are associated with those outcomes. Understanding these patterns can inform ongoing efforts to build teaching capacity in the region.

The objective of this study was to describe IMEP’s medium- to long-term outcomes across six annual cohorts from 2019 to 2024 based on an alumni survey, focusing on project implementation, dissemination of educational innovations, and professional accomplishments. A secondary objective was to examine post-training outcomes across participating institutions and faculty seniority levels. We interpreted findings through the lens of transfer of training theory to contextualize outcomes in relation to partnership-based program design and institutional context.

## Methods

### Study design and setting

This cross-sectional descriptive study evaluated the outcomes of the International Medical Educators Program (IMEP). IMEP is conducted by the University of Chicago in collaboration with teaching hospitals in China.

### Program description

IMEP is a 4-month faculty development initiative designed to strengthen participants’ skills in curriculum design, effective teaching, and educational leadership (Table 1). The program is delivered through a combination of in-person and online learning, totaling approximately 50 h of face-to-face instruction (including Zoom). The curriculum includes sessions on curriculum development, learning theory, small group teaching, lecture and slide design, growth mindset, adaptive behavior in medicine, outpatient teaching skills, teaching in time limited settings, bedside teaching skills, feedback, coaching, and professional identity formation. Participants also visit a simulation center, complete an OSTE (objective structured teaching exercise) with standardized learners, and tour the Accreditation Council for Graduate Medical Education headquarters. A core component of the program is the design and implementation of a curricular project, guided by faculty mentorship throughout the duration of the program [13].

**Table 1:** Structure of the International Medical Educators Program (IMEP)

Program phase	Timing	Learning focus	Learning activities	Transfer-relevant design features
In-person Intensive	2 weeks	Foundational skills in curriculum design, teaching, and educational leadership	Workshops on curriculum development; lecture and slide design; teaching in time-limited and bedside settings; small group teaching; feedback and coaching skills; simulation center visit; objectives structured teaching exercise (OSTE); exposure to U.S. graduate medical education accreditation systems	Skill acquisition through experiential learning; shared conceptual frameworks; immediate feedback; development of peer and mentor relationships
Longitudinal Online Follow-up	7 sessions over 3.5 months	Application of learning to local educational contexts and project implementation	Mentored project development and refinement; project homework; peer discussion	Reinforcement and application of skills; accountability for project progress; mentorship and peer support

IMEP incorporates several design features informed by transfer of training frameworks. To increase trainee motivation and institutional readiness, participants must obtain letters of support from their supervisors and be nominated by their institutions. After completion of the program, in-person site visits to alumni hospitals by Chicago IMEP faculty leaders – a distinctive element made possible through long-term institutional partnerships – enhance continued mentorship and engagement. Expectations of project implementation, combined with mentorship and follow-up site visits, support continued application of new skills.

During 2020 to 2023, the program was conducted entirely online due to COVID-19 travel restrictions, reflecting the continuity of the partnership despite disruptions [13]. Starting 2024, the 2-week in-person component resumed, followed by 3 months of online mentoring.

### Participants and data collection

Between 2019 and 2024, IMEP enrolled 86 faculty participants from primarily four Chinese academic medical centers: Renmin Hospital and Zhongnan Hospital (both affiliated with Wuhan University), PUMCH, and a small number from First Affiliated Hospital of Sun Yat-Sen University (FAH-SYSU).

All 86 clinicians who participated in IMEP from 2019 to 2024 were invited to complete a follow-up survey. The class of 2025 was ongoing at the time of the survey and therefore excluded. As this was a census survey of all IMEP participants, formal sample size calculations were not performed. Survey administration occurred 1–5 years post-training. The survey was developed by the study team based on IMEP program objectives, prior literature on faculty development outcomes, and the higher levels of Kirkpatrick’s evaluation model. The bilingual (English/Chinese) survey was created in Redcap and distributed through WeChat on August 5, 2025. Weekly reminders were sent, and the survey closed August 23, 2025. Participation was voluntary and survey responses were anonymous. Completion and submission of the survey implied informed consent to participate.

The survey assessed training year, institutional affiliation, titles, project topics and post-training outcomes. Outcomes were assessed according to the higher levels of impact described by Kirkpatrick and Kirkpatrick’s model [10].

- Level 3 (behavior): application of learning to educational practice, including project implementation, initiation of new projects, dissemination of innovations, national or regional presentations, and sharing of IMEP learning with colleagues.
- Level 4 (results): participant-reported professional and organizational outcomes, including teaching awards, promotion based on educational work, new leadership positions in education, impact of project, curricular reform, publications in education, and institutional support for educators.

Response categories were primarily yes/no with optional free-text fields for project descriptions and titles. Open-ended responses describing institutional changes were reviewed and summarized into thematic categories by the research team. Survey responses were reviewed for completeness. Incomplete surveys were excluded.

### Variable definitions

China maintains parallel academic and clinical promotion systems, and titles were collapsed into two categories for analysis: senior (professor, chief physician, or department director/deputy director) and junior (associate professor, deputy chief physician, attending, or teaching secretary).

For institutional comparisons, Wuhan University-affiliated hospitals (Renmin Hospital and Zhongnan Hospital) were combined into a group, reflecting their shared university affiliation and similar function as leading provincial teaching hospitals. PUMCH was analyzed as a distinct institutional group due to its role as a leading national academic medical center. Participants from FAH-SYSU were reported descriptively due to the small sample size and not included in analyses.

### Statistical analysis

Survey responses were summarized using descriptive statistics. The response rate was calculated as the number who completed the survey divided by the number of eligible participants. Frequencies and percentages were calculated for respondent characteristics, current faculty titles, and post-training outcomes. Pearson’s chi square test was used to compare (1) faculty seniority distributions between PUMCH and Wuhan University-affiliated hospitals and (2) differences in post-training outcomes between these institutions. Since only three respondents came from FAH-SYSU, this group was excluded from statistical comparisons.

To assess whether institutional differences were explained by faculty seniority, multivariable binary logistic regression analyses were conducted for each dichotomous post-training outcome. Institution (PUMCH vs Wuhan University hospitals) and seniority (senior vs junior) were entered as independent variables. Adjusted odds ratios (aORs) with 95% confidence intervals (CI) were reported.

*P*-values < 0.05 were considered statistically significant. Analyses were conducted in SPSS version 31.0.0.0 (IBM Corp).

This study was reviewed by the University of Chicago Institutional Review Board and determined to be exempt (IRB25-1180).

## Results

Sixty-three IMEP alumni responded to the survey; three responses were excluded (two incomplete and one from the 2025 cohort), yielding 60 responses and a 69.8% response rate. Respondents came from all cohort years from 2019 to 2024, with the largest groups from 2020 (23.3%) and 2019 (18.3%) (Table 2). Response rates by cohort year ranged from 55.6% to 100%. Thirty-two (53.3%) respondents were affiliated with Wuhan University hospitals and 24 (40%) with PUMCH; three were from FAH-SYSU, and one respondent was not affiliated with a hospital. Since only three respondents came from FAH-SYSU, comparisons focused on PUMCH and Wuhan University-affiliated hospitals.

**Table 2:** Characteristics of survey respondents (*N* = 60)

Respondent characteristic	n (%)	% of year’s cohort
Training year		
2019	11 (18.3%)	100%
2020	14 (23.3%)	70.0%
2021	10 (16.7%)	55.6%
2022	8 (13.3%)	57.1%
2023	8 (13.3%)	61.5%
2024	9 (15.0%)	100.0%
Institution		
PUMCH	24 (40.7%)	
Wuhan Uni. Hospitals	32 (54.2%)	
FAH-SYSU	3 (5.1%)	

Total for “Institution” is 59 because one respondent was not affiliated with a hospital

Current title ranks were available for all survey participants with the exception of one respondent not affiliated with a hospital. Two-thirds of PUMCH respondents held senior titles compared to one-quarter of Wuhan respondents (66.7% vs 25.0%, *p =* 0.002) (Table 3). This imbalance in distribution informed the decision to adjust for faculty rank in multivariable analysis.

**Table 3:** Current faculty seniority by institution (*N* = 56)

Title category	PUMCH (*n* = 24)	Wuhan Uni. (*n* = 32)
Senior	16 (66.7%)	8 (25.0%)
Junior	8 (33.3%)	24 (75.0%)

Respondents from FAH-SYSU (n- = 3) and one not affiliated with a hospital were excluded from this table. Pearson chi-square test on the distribution of faculty seniority by institution was statistically significant (*p =*.002)

### Description of IMEP projects

Survey respondents described a wide range of project topics spanning multiple specialties. Projects clustered into several broad categories. Simulation-based and procedural skill training was common, including airway management and neonatal resuscitation. Instructional innovations such as blended learning, flipped classroom, and bedside teaching were also frequent topics. Specialty-specific projects addressed educational needs within clinical disciplines. Communication-focused projects addressed doctor-patient communication and communication of diagnostic uncertainty. Additional projects addressed transitions and training structure, as well as faculty development.

Several large-scale initiatives stood out for their scope. These included the development of a competency-based evaluation system for obstetrics and gynecology spanning undergraduate and graduate medical education, creation of a multi-year rare diseases curriculum and textbook, and an “Afternoon Tea” growth mindset and wellness series. See Appendix for a full list of projects.

### Overall outcomes and professional achievements of IMEP alumni

With respect to Kirkpatrick level 3 (behavior) outcomes, 36 participants (60%) reported implementing their original project at their home institution, and 38 (63.3%) reported developing new teaching projects since IMEP (Table 4). A total of 48 (80%) implemented at least one project, and half (50%) shared their projects with others more broadly (e.g. through workshops). A majority (75%) shared what they learned through IMEP with other educators and colleagues, and fourteen (23.3%) presented their educational work at regional or national conferences.

**Table 4:** Overall Outcomes of IMEP Alumni (*N* = 60)

Outcome	n	%
Level 3 (behavior)		
Project implemented	36	60
New teaching projects since IMEP	38	63.3
Shared projects externally	30	50
National/regional presentation	14	23.3
Shared IMEP learning with colleagues	45	75
Level 4 (results)		
Teaching award	30	50
Promotion (education-based)	15	25
New position in med ed	16	26.7
Curriculum reform	30	50
Impact at local/regional/national level	22	36.7
Publication/textbook	30	50
Institutional support for educators	41	68.3

For level 4 (results) outcomes, 30 participants (50%) received a teaching award, 15 (25%) were promoted based on educational work, and 16 (26.7%) received a new position in medical education. Nearly half (48.3%) published peer-reviewed articles or textbooks, and 30 (50%) reported leading curricular reform. Twenty-two (36.7%) reported that their project had a substantial local, regional, or national impact. Since participation in IMEP, 68.3% noticed positive changes in how their institutions supported community among medical educators; no participants reported negative changes. In open-ended responses to this item, participants described establishment of formal teaching development centers, new policies and incentives for educators, expanded training and exchange opportunities, integration of simulation and technology, and increased participation in international collaborative networks.

### Institutional comparisons

Comparisons between IMEP alumni from PUMCH and Wuhan University-affiliated hospitals are shown in Table 5. Across most outcomes, differences between institutions did not reach statistical significance. The only unadjusted difference observed was a higher likelihood of initiating new teaching projects among PUMCH respondents compared with Wuhan University-affiliated respondents (79.2% vs 53.1%, *p =* 0.044). Other outcomes, including conference presentations, promotion, and reported project impact, showed similar trends but did not reach statistical significance.

**Table 5:** Outcomes by institution

Outcome	PUMCH (*N* = 24), n (%)	Wuhan Uni. (*N* = 32), n (%)	p-value (unadj.)	Adjusted OR (95% CI)	*p*-value (Adj.)
Project implemented	16 (66.7%)	17 (53.1%)	0.308	1.50 (0.45–4.97)	0.509
New teaching projects since IMEP	19 (79.2%)	17 (53.1%)	0.044^*^	3.31 (0.89–12.30)	0.075
Shared projects externally	14 (58.3%)	13 (40.6%)	0.189	1.92 (0.59–6.24)	0.277
National/regional presentation	8 (33.3%)	4 (12.5%)	0.060	3.78 (0.86–16.71)	0.079
Shared IMEP learning with colleagues	19 (79.2%)	23 (71.9%)	0.533	1.62 (0.41–6.41)	0.494
Teaching award	14 (58.3%)	14 (43.8%)	0.280	1.76 (0.54–5.69)	0.348
Promotion (education-based)	8 (33.3%)	4 (12.5%)	0.060	4.88 (1.06–22.51)	0.042^**^
New position in med ed	8 (33.3%)	7 (21.9%)	0.338	1.81 (0.49–6.75)	0.374
Curriculum reform	14 (58.3%)	13 (40.6%)	0.189	1.67 (0.51–5.40)	0.394
Impact at local/regional/national level	11 (45.8%)	7 (21.9%)	0.057	1.65 (0.44–6.13)	0.458
Publication/textbook	13 (54.2%)	13 (40.6%)	0.315	1.71 (0.53–5.52)	0.373
Institutional support for educators	16 (66.7%)	24 (75%)	0.495	0.75 (0.21–2.71)	0.661

^*^p < 0.05 for group differences by Pearson’s chi-square test

^**^p < 0.05 for adjusted odds ratio from binary logistic regression controlling for current faculty seniority

After adjusting for both institutional affiliation and faculty seniority, the direction of associations remained similar. PUMCH respondents were more likely to report promotion based on educational work (adjusted OR 4.88, 95% CI 1.06–22.51, *p =* 0.042). PUMCH respondents also remained more likely to initiate new teaching projects, although this association did not reach statistical significance (*p =* 0.075). Senior faculty were more likely to report project impact after adjusting for institutional affiliation (adjusted OR 5.28, 95% CI 1.39–20.03, *p =* 0.014).

## Discussion

### Key findings

We found that faculty from multiple specialties across all participating hospitals demonstrated substantial educational outcomes including project implementation, curricular reform, promotion, new positions in medical education, scholarly dissemination, and teaching awards. Two-thirds of participants reported noticing positive changes in institutional support for educators since their participation in IMEP, with no reports of negative change. These findings suggest that a longitudinal, partnership-based faculty development model is associated with sustained educational impact across diverse institutional settings.

These findings can be interpreted through transfer of training theory, which emphasizes that the application of learning to practice depends on trainee characteristics, training design, and the work environment [2, 7]. IMEP was designed to engage all three domains: trainee characteristics through motivation and confidence, training design through mentored experiential learning, and work environment through institutional engagement.

### Alignment with established models of faculty development

IMEP outcomes parallel those reported in other longitudinal programs that deliberately include features which facilitate transfer [14]. The Macy Faculty Scholars Program showed that protected time, mentorship, and an engaged community of practice were crucial factors in faculty growth and academic productivity [8]. These elements strengthen both the training design and work environment in Baldwin and Ford’s model. Similarly, the McGill Teaching Scholars Program for Educators in the Health Sciences, which uses longitudinal, mentored, project-based learning, found most participants assumed new educational roles, maintained improved teaching practices, and disseminated their work through conferences and publications [18]. These findings reinforce that enhancing training design enables faculty to translate educational principles into durable institutional change.

IMEP demonstrates that even within a shorter 4-month time frame, programs that integrate longitudinal engagement, mentorship, and project-based learning may be associated with meaningful outcomes. Alumni balanced project implementation alongside clinical duties, illustrating the feasibility of achieving educational impact even without the protected time offered by programs such as Macy, when using strategies shown to enhance transfer to practice [22]. At the same time, IMEP’s impact appears to be shaped not only by program design, but also by the broader organizational context created through long-standing collaboration between the University of Chicago and its partner hospitals.

### Institutional partnerships and organizational context

The decade-plus relationship between University of Chicago and partner hospitals represents an organizational context relevant to transfer. Longstanding institutional partnerships may strengthen the work environment by promoting trust, continuity, and supervisor engagement. They may also contribute to a cultural shift that values education alongside clinical and research missions.

A distinctive feature of IMEP is the opportunity for continued engagement through voluntary post-training site visits by Chicago faculty. These visits, made possible by longstanding institutional relationships, extend mentorship beyond the formal training period and help sustain motivation and project momentum. Evidence from other settings supports this approach: Goldman et al. demonstrated that participants receiving post-training structured mentorship implemented 23% more of their planned initiatives, including more leadership and curricular projects [6]. IMEP’s model of maintaining informal post-training connections aligns with these findings. The continuity of these institutional partnerships may also increase accountability and expectations for project implementation, reinforcing transfer through social norms [4]. Such partnerships may be particularly effective in international contexts where cultural and institutional environments differ.

Project completion rates were comparable to or exceeded other programs [1, 16]. Implementation rates were highest during the initial COVID-19 years, suggesting that other factors such as greater flexibility in clinical duties and the rapid shift to online teaching modalities facilitated project execution.

### Variation in institutional work environments

We found that the majority of participants completed their education reform projects with few consequential differences between institutions, suggesting that the IMEP model can be applied to varied institutional settings. Even though PUMCH respondents showed higher rates across most outcomes, only promotion based on educational work remained significant in adjusted analyses. This may reflect variations in how institutions recognize and support educational innovation. For example, PUMCH is designated a national medical education demonstration center and provides clearer pathways and protected time for faculty advancement in education, whereas many other institutions continue to emphasize scientific publications as the main promotion criteria. However, given the modest sample size and wide confidence intervals, these institutional differences should be interpreted cautiously.

The breadth of outcomes observed across hospitals suggests that faculty development programs can be effective across diverse institutional settings, and that concentrating investment solely in elite centers may not be necessary to achieve impact. Participants’ reports of increased institutional support for educators provide additional context for interpreting these outcomes. Respondents described new teaching centers, policies, and educational technologies at their institutions. These changes may reflect broader national reform trends, institutional responses to faculty who returned with new ideas and energy, or heightened awareness among participants themselves [19]. Regardless of attribution, these perceptions suggest that IMEP alumni are increasingly practicing in contexts that value educational work. Future iterations of IMEP and similar programs might explore addressing additional work environment factors, including more structured post-training mentorship and deliberate engagement with departmental leaders to clarify expectations and better align projects with institutional priorities.

### Limitations and future directions

This study has several limitations. Data were self-reported and cross-sectional, without verification from curricula vitae and institutional records. There was no comparison group, and response bias cannot be excluded. Although this study surveyed the entire eligible alumni population, IMEP is a relatively small faculty development program. Institutional comparisons were limited by modest cohort sizes and resulting wide confidence intervals. Nevertheless, the 70% response rate and multi-institutional representation provide a valuable snapshot of medium- to long-term outcomes. While we interpret findings through a transfer of training lens, we did not include measures of work environment such as supervisor and peer support. Future directions include examining which environmental supports most strongly predict sustained transfer across institutional contexts.

## Conclusions

Findings from IMEP suggest that longitudinal, partnership-based faculty development is associated with sustained educational and professional outcomes across diverse institutional contexts in China. Participants implemented innovative educational projects, advanced as scholars and leaders, and contributed to institutional change, with broadly comparable impact across participating hospitals. Longstanding institutional relationships may have supported continued application of learning beyond the formal training period by creating continuity and accountability, and ongoing engagement. These findings contribute to the growing body of evidence that such programs not only strengthen individual educators but also build institutional capacity in health professions education.

## Data Availability

The datasets used during the current study are available from the corresponding author on reasonable request.

## Appendix

### IMEP Participants Project Titles (2019-2024)

Section A. Original IMEP Projects

#### Simulation-based and procedural skill training

- Urology simulation
- Balloon mask ventilation and endotracheal intubation techniques
- Pediatric lumbar puncture
- Enhancing emergency management skills of 2^nd^ year obstetrics residents
- Application of simulation teaching to improve residents’ workplace competency
- Neonatal resuscitation
- Intubation and airway management
- Laparoscopic basic skills and anatomy training
- Application of scenario simulation combined with Mini-CEX in clinical reasoning training and evaluation of endocrine and metabolic diseases
- Development of surgical skills training system based on hierarchical progressive training
- Application of BOPPPS teaching mode in lumbar puncture teaching
- Airway management in critically ill patients
- Training in renal biopsy for young nephrology physicians
- Evaluation of the application effect of electronic package in the clinical skills teaching of pediatrics
- Neonatal resuscitation program for pediatric residents
- Competency training for critical emergency diseases: a scenario-based simulation curriculum for emergency residents
- Imaging post-processing training course for radiology residents
- Application of a digital integrated teaching platform in practical skills training for residents preparing for physician licensing

#### Instructional innovations

- Building a virtual patient database
- Brief feedback
- Application of SPOC-supported blended courses in obstetrics and gynecology teaching
- Critical online open course
- A new pattern of blended learning of clinical skills based on flipped classroom and self-directed clerkship
- Bedside teaching
- Discussion on course reform and optimization model of quality education in obstetrics and gynecology– peer teaching
- Applied a PBL + CBL-based Think-Pair-Share (TPS) model for GME clinical reasoning and teamwork
- Visual abstracts for GME
- Cranial nerve anatomy learning in neurosurgery residents
- Bedside teaching of geriatrics for internal medicine residents

#### Specialty-specific curriculum development

- Ob gyn clerkship outpatient education
- Developing a curriculum on improving self-directed learning
- Modern geriatrics curriculum
- Program of emerging Infectious Diseases and hospital infection control for medical students after the outbreak of COVID-19
- Risks of complications and unplanned reoperation in cataract patients with residents’ participation
- Robotic pancreatic surgery
- Coronary Heart Disease Basic to Clinical
- Comprehensive assessment in Geriatrics
- Sleep medicine
- Emergency palliative care
- EPA framework–based general surgery training curriculum development
- Application of Kern’s 6-Step Curriculum Development in teaching sudden cardiac death
- Diagnostic reasoning of rare kidney disease (RKD) for residents
- Implementation of Kern's Six Steps in designing teaching program on NPC target volume delineation

#### Communication-focused projects

- Six steps for doctor-patient communication
- Doctor-patient communication course
- How to deliver effective morning report in morning shifts for first-year residents
- Training course on fetal genetic disease counseling for obstetricians
- Training residents to communicate diagnostic uncertainty in the emergency department
- Interpretation and communication of pediatric test results

#### Transitions and training structure

- A transition curriculum for medical students preparing competency during night shift duties
- Time control strategies for resident trainees
- Application of a stepwise weekly goal-based internship syllabus in obstetrics and gynecology training

#### Faculty development

- A course on medical innovation and technology transfer for junior faculty
- Kern’s 6-Step teaching method
- IMEP
- Academic research

#### Other

- Curriculum for residents

Section B. New Teaching Projects Initiated After IMEP

#### Simulation-based and procedural skill training

- Robotic intestinal anastomosis course
- Training to simulation instructors
- Video resource library for teaching pediatric bone marrow aspiration to undergraduates
- Piloting scenario simulation combined with a modified BOPPPS model in clinical reasoning training
- Application of virtual simulation seminar-based teaching in obstetrics and gynecology clinical practice

#### Instructional innovations

- Medical Imaging and Artificial Intelligence
- Establishment of an AI-assisted teaching aid for professional master’s degree students in clinical laboratory science
- Development and application of a student portfolio management system based on surgical skill operation videos
- Establishment of an integrated ideological and political education case database
- Blended learning reform of current teaching courses
- The application of AI in medical education (large language models, ASR, TTS)
- Implementing a hierarchical progressive model to conduct clinical teaching on cardiology knowledge and skills for professional master’s degree students
- OSTE for residents in internal medicine department

#### Specialty-specific curriculum development

- Online course on common gynecologic endocrine diseases
- Construction of a competency-based evaluation system for obstetrics and gynecology education across all stages
- Rare Diseases Curriculum and textbook project
- Training course on the interpretation of pathogenicity of genetic variants for genetic laboratory data analysts
- Standardized training on chronic kidney disease and renal failure
- Integrated curriculum model for pediatric urinary system diseases
- Pediatrics online–offline blended “First-Class” undergraduate course
- Application of multidisciplinary integration in obstetrics and gynecology teaching under the“New Medical Science” initiative
- Establishment of an advanced clinical skills training and assessment system for young physicians in internal medicine

#### Communication-focused projects

- Application effect of structured interviewing techniques in obstetrics and gynecology clinical clerkship teaching
- Survey and analysis of non-technical skills among anesthesiology residents

#### Transitions and training structure

- Establishment and implementation of a continuous competency-based assessment system for surgical residents
- Building a Bridging Training System for Surgical Residency Education
- Final examination and skills assessment for resident physicians

#### Other

- Small-scale programs in resident training and medical English teaching
- A new assessment according to the curriculum
